# Aspartate α-decarboxylase a new therapeutic target in the fight against *Helicobacter pylori* infection

**DOI:** 10.3389/fmicb.2022.1019666

**Published:** 2022-11-07

**Authors:** Kareem A. Ibrahim, Mona T. Kashef, Tharwat R. Elkhamissy, Mohammed A. Ramadan, Omneya M. Helmy

**Affiliations:** ^1^Department of Microbiology and Immunology, Faculty of Pharmacy, Egyptian Russian University, Cairo, Egypt; ^2^Department of Microbiology and Immunology, Faculty of Pharmacy, Cairo University, Cairo, Egypt

**Keywords:** *Helicobacter* *pylori*, aspartate α-decarboxylase, broad-spectrum, malonic acid, drug target

## Abstract

Effective eradication therapy for *Helicobacter pylori* is a worldwide demand. Aspartate α-decarboxylase (ADC) was reported as a drug target in *H*. *pylori*, in an *in silico* study, with malonic acid (MA) as its inhibitor. We evaluated eradicating *H*. *pylori* infection through ADC inhibition and the possibility of resistance development. MA binding to ADC was modeled *via* molecular docking. The minimum inhibitory concentration (MIC) and minimum bactericidal concentration (MBC) of MA were determined against *H*. *pylori* ATCC 43504, and a clinical *H*. *pylori* isolate. To confirm selective ADC inhibition, we redetermined the MIC in the presence of products of the inhibited enzymatic pathway: β-alanine and pantothenate. HPLC was used to assay the enzymatic activity of *H*. *pylori* 6x-his tagged ADC in the presence of different MA concentrations. *H*. *pylori* strains were serially exposed to MA for 14 passages, and the MICs were determined. Cytotoxicity in different cell lines was tested. The efficiency of ADC inhibition in treating *H*. *pylori* infections was evaluated using a Sprague–Dawley (SD) rat infection model. MA spectrum of activity was determined in different pathogens. MA binds to *H*. *pylori* ADC active site with a good docking score. The MIC of MA against *H*. *pylori* ranged from 0.5 to 0.75 mg/mL with MBC of 1.5 mg/mL. Increasing β-alanine and pantothenate concentrations proportionally increased MA MIC. The 6x-his tagged ADC activity decreased by increasing MA concentration. No resistance to ADC inhibition was recorded after 14 passages; MA lacked cytotoxicity in all tested cell lines. ADC inhibition effectively eradicated *H*. *pylori* infection in SD rats. MA had MIC between 0.625 to 1.25 mg/mL against the tested bacterial pathogens. In conclusion, ADC is a promising target for effectively eradicating *H*. *pylori* infection that is not affected by resistance development, besides being of broad-spectrum presence in different pathogens. MA provides a lead molecule for the development of an anti-helicobacter ADC inhibitor. This provides hope for saving the lives of those at high risk of infection with the carcinogenic *H*. *pylori*.

## Introduction

*Helicobacter pylori* is the primary cause of peptic ulcer, besides being classified as a class I carcinogen by the World Health Organization. Infection with *H*. *pylori* is associated with both intra-gastric and extra-gastric disorders. Despite the extensive research during the last three decades, no effective vaccine against *H*. *pylori* is available (Robinson and Atherton, 2021). Antibiotics are used for the clinical management of *H*. *pylori* infections (Xu et al., 2021). However, poor patients’ compliance to the long and complex treatment regimens and the fast-paced antibiotic resistance development magnified by the stalled development of new anti-helicobacter agents have posed a global threat (Ventola, 2015; Abadi, 2016). This requires an urgent intervention to propose new treatments for *H*. *pylori* infections (Xu et al., 2021).

The availability of the genomic sequences of pathogenic bacteria has provided huge data that can be used for identifying potential drug and vaccine targets through subtractive genomic, proteomic or transcriptomic approaches (Nandode et al., 2012; Yan and Gao, 2020). Using a subtractive proteomic approach, we previously identified 17 essential targets in *H*. *pylori* with 42 possible Drugbank ligands, of which several small organic acids were potential ligands for many of the retrieved essential targets (Ibrahim et al., 2020). These molecules have a well-known antibacterial activity, such as (S)-3-phenyllactic acid (Mu et al., 2012), citric acid (Adamczak et al., 2020), malonic acid (Feng et al., 2010), dipicolinic acid (Jadamus et al., 2005), and D-tartaric acid (Hu et al., 2019; Coban, 2020).

Aspartate α-decarboxylase (ADC) enzyme was proposed as an essential drug target, conserved in *H*. *pylori* and more than 200 common pathogens (Ibrahim et al., 2020), suggesting it as broad-spectrum target. ADC catalyzes the bacterial alpha decarboxylation of L-aspartate into β-alanine, required for pantothenate production (the ionized form of pantothenic acid or vitamin B5). Pantothenate is the precursor of CoA (Song et al., 2015; Zhang et al., 2015), which is an essential cofactor for many enzymes in almost all living organisms. Nearly 9% of the 3,500 enzymatic activities identified in the Braunschweig enzyme database use CoA as a cofactor in the metabolism of fats, carbohydrates, and proteins, as well as energy production (Spry et al., 2008). Disruption of the genes/enzymes in CoA biosynthesis can lead to lethal phenotypes (Walia et al., 2009). ADC was identified as a *Mycobacterium tuberculosis* drug target inhibited by pyrazinamide (Gopal et al., 2020). Malonic acid was proposed as a potential inhibitor of the *H*. *pylori* ADC enzyme (Ibrahim et al., 2020).

In this study, we confirmed ADC as a promising drug target for eradicating *H*. *pylori* infections. Besides being conserved in many pathogenic species, no resistance development to ADC inhibition was detected in *H*. *pylori* following 14 serial passages. The possible utilization of malonic acid as an ADC inhibitor is also proposed, where malonic acid can be used as a lead molecule for developing ADC inhibitors.

## Materials and methods

### Bacterial strains and culture conditions

*Helicobacter pylori* ATCC 43504 and a clinical *H*. *pylori* isolate (HPM001) from the culture collection of the Department of Clinical Pathology, Faculty of Medicine (Kasr El-Aini), Cairo University, Cairo, Egypt, were used in the study. *H*. *pylori* strains were stored in brain heart infusion broth (MAST, United Kingdom) containing 10% fetal bovine serum (FBS) (Sigma-Aldrich, Germany) and 20% glycerol at −70°C. When needed, and unless otherwise stated, *H*. *pylori* was subcultured on Columbia agar base (LabM, United Kingdom) containing 5% sheep blood and DENT supplement (Oxoid, United Kingdom), and incubated at 37°C for 72 h under microaerophilic conditions (5% O_2_, 10% CO_2_, and 85% N_2_ at 95% humidity) using CampyGen paper sachets (Oxoid, United Kingdom; Piccolomini et al., 1997) or a candle jar (Sudhakar et al., 2008).

*Escherichia coli* DH5α and *E*. *coli* BL21 were used in cloning and expression experiments. *Acinetobacter baumannii* ATCC 19606, *Burkholderia cenocepacia* ATCC BAA-245, *E*. *coli* ATCC 25922, *Enterococcus faecium* ATCC 27270, *Enterococcus faecalis* ATCC 19433, *Klebsiella pneumoniae* ATCC 10031, *Pseudomonas aeruginosa* ATCC 27856, and *Staphylococcus aureus* ATCC 25923 were used in testing the spectrum of activity. They were stored in Muller-Hinton broth (Oxoid, United Kingdom) containing 20% glycerol at −70°C. When needed, they were subcultured on Lauria Bertani (LB) agar (LabM, United Kingdom) and incubated at 37°C for 18 h. The culture media were supplemented with 50 μg/mL ampicillin if required.

### Molecular docking of malonic acid to the active site of ADC enzyme

Before performing the modeling study, the amino acid sequences of the ADC enzyme (about 50 sequences) from *H*. *pylori* 26695 (PDB ID: 1UHE; used in docking study), *H*. *pylori* ATCC 43504 (the standard strain used in this study) and from randomly selected *H*. *pylori* strains were downloaded from the National Center for Biotechnology Information (NCBI) and analyzed by multiple sequence alignment, using Clustal Omega (Madeira et al., 2022). The molecular docking study of malonic acid to the binding site of ADC was done at the Micro-analytical Unit, Molecular Modelling Laboratory, Faculty of Pharmacy, Cairo University, Cairo, Egypt. The modeling studies were performed using Molecular Operating Environment (MOE, 2015.10). All minimizations were performed with MOE until a root mean squared distance gradient of 0.05 kcal.mol^−1^ Å^−1^ was reached using MMFF94x force field, and the partial charges were automatically calculated. The X-ray crystallographic structure of the *H*. *pylori* ADC enzyme (PDB ID: 1UHE) was downloaded from the Protein Data Bank. Ligands not involved in binding and water molecules were removed from each co-crystallized enzyme. The protein was prepared for the docking study using Protonate 3D protocol in MOE with default options. The co-crystalized ligand (N-2-(2-amino-1-methyl-2-oxoethylidene) asparaginate) was used to define the binding site for docking. Triangle Matcher placement method and London ΔG scoring function were used for docking.

Docking setup was first validated by self-docking the co-crystallized ligand in the vicinity of the enzyme’s binding site. The docking of the enzyme’s natural ligand (aspartate) was performed to expose its intermolecular interactions with the active binding site. The validated setup was then used to predict the malonate-receptor interactions at the ADC binding site.

### Determination of malonic acid minimum inhibitory concentration

The MIC of malonic acid was determined by agar dilution and broth microdilution methods, against *H*. *pylori* ATCC 43504 and *H*. *pylori* HPM001 strains. Malonic acid (Loba, India) was dissolved in distilled water to the desired concentration, sterilized by a 0.22 μM syringe filter, and used within 72 h of preparation. Inocula for MIC testing were prepared by suspending colonies in saline to reach an optical density equivalent to 2.0 McFarland turbidity standard (approximately 1×10^7^ to 1×10^8^ CFU/mL) (CLSI, 2015). The culture media used in MIC determination were freshly prepared, according to the manufacturer’s instruction, and supplemented with 1% yeast extract (LabM, United Kingdom) to enhance bacterial growth (Walsh and Moran, 1997).

#### Agar dilution method

Determination of MIC by the agar dilution method was performed according to the clinical and laboratory standards institute (CLSI) guidelines (CLSI, 2015). Briefly, 2 μL of the inoculum (containing 1×10^4^ CFU) was spotted on the surface of Muller-Hinton agar (Oxoid, United Kingdom) plates supplemented with 5% sheep blood and containing the specified concentration of malonic acid (6 to 0.19 mg/mL) followed by incubation at 37°C for 72 h, under microaerophilic conditions. The MIC was the lowest concentration of malonic acid that completely inhibited visible bacterial growth (CLSI, 2015). The experiment was done in triplicates.

#### Broth microdilution method

MIC determination by broth microdilution method was carried out in 96-well round-bottom micro-titre plates. Each well contained 100 μL of brucella broth (Conda, Spain) containing 10% FBS. Malonic acid solution (12 mg/mL) was two-fold serially diluted in the broth-FBS mixture to get concentrations ranging from 6 to 0.19 mg/mL. The bacterial inoculum was diluted 1:10, and 10 μL of the diluted inoculum was transferred to each well to contain 5 × 10^5^ CFU/mL followed by incubation at 37°C for 72 h under microaerophilic conditions. The MIC was the lowest concentration of malonic acid completely inhibiting the visible growth of the tested organism. The experiment was carried out in triplicates.

### Determination of the minimum bactericidal concentration of malonic acid

The MBC of malonic acid against both *H*. *pylori* ATCC 43504 and *H*. *pylori* HPM001 was determined, according to Moraes and colleagues (Moraes et al., 2021). Briefly, following MIC determination by broth microdilution method, 10 μL were transferred from wells showing no visible growth onto the surface of Muller-Hinton agar plate supplemented with 5% sheep blood and incubated at 37°C for 72 h, under microaerophilic conditions. The MBC was the lowest concentration that failed to show bacterial growth on the agar plate.

### Evaluation of ADC inhibition by malonic acid

#### Determination of the MIC of malonic acid in presence of β-alanine and pantothenate

β-alanine and pantothenate, the downstream products of the enzymatic pathway (EC: 4.1.1.11) potentially inhibited by malonic acid, were used to confirm the selective inhibition of *H*. *pylori* ADC enzyme by malonic acid. The MIC of β-alanine (Sigma-Aldrich, Germany) and pantothenate (Loba Chemie, India) against *H*. *pylori* ATCC 43504 and *H*. *pylori* HPM001 were determined by agar dilution and broth microdilution methods, as described earlier. The MIC of malonic acid against *H*. *pylori* ATCC 43504 and *H*. *pylori* HPM001 was determined in the absence and presence of increasing sub-inhibitory concentrations of β-alanine (0.6–560 mM) or pantothenate (0.25–228 mM). Experiments were done in triplicates.

#### Assessment of recombinant *Helicobacter pylori* 6xHis-tagged ADC activity in presence of malonic acid

##### Cloning and expression of *Helicobacter pylori* ADC enzyme

The genomic DNA of *H*. *pylori* ATCC 43504 was extracted using GeneJet Genomic DNA Purification Kit (Thermo Fisher Scientific, Lithuania). Primers used in the study were supplied by Macrogen, Korea, and are listed in Supplementary Table S1. The *pan*D gene was amplified using KI001 and KI002 primers; The polymerase chain reaction (PCR) products were digested by *Eco*RI/*Xho*I enzymes (Puregene, Genetix, India) and ligated using T4 DNA ligase (Takara, Japan) with the similarly digested pET-22b(+) plasmid (Merck, Germany). The recombinant plasmid (RecPl) was transformed into chemically competent *E*. *coli* DH5α cells (Sambrook and Russel, 2001). Clones were selected on LB agar plates containing 50 μg/mL ampicillin; the LB agar plates were incubated at 37°C for 24 h, and the obtained colonies were screened by PCR using combinations of the following primers; KI001, KI002, KI003 (Yang et al., 2020), and KI004 (Yang et al., 2020).

RecPl from successful clones was extracted using QIAprep Spin Miniprep kit (Qiagen, Germany) and transformed into chemically competent *E*. *coli* BL21. Overnight culture of *E*. *coli* BL21/RecPl was subcultured in LB broth containing 50 μg/mL ampicillin and incubated at 37°C with shaking (250 rpm) to reach an optical density of approximately 0.6 at 600 nm. Protein expression was induced by using 0.75 mM isopropyl β-D-1-thiogalactopyranoside (IPTG) (Scharlau, Spain), and incubation at 37°C for 4 h with shaking at 200 rpm. Cells were harvested by centrifugation at 6000 rpm for 10 min at 4°C and resuspended in binding buffer (50 mM sodium phosphate buffer, 500 mM NaCl and 100 mM imidazole, pH = 7.4). The crude extract, of IPTG-induced *E*. *coli* BL21/RecPl in binding buffer, was prepared by sonication for 30 min on ice, followed by centrifugation at 6000 rpm for 20 min at 4°C and filtered using 0.45 μM syringe filter (Mo et al., 2018). 6xHis-tagged ADC protein purification was performed using Ni-NTA spin columns (Qiagen, Germany), according to the manufacturer’s instructions. The purified protein was analyzed using sodium dodecyl sulphate-polyacrylamide gel electrophoresis (SDS-PAGE) and its concentration was determined by BCA protein assay kit (Novagen, Germany), according to the manufacturer’s instructions.

##### Assay of *Helicobacter pylori* 6xHis-tagged ADC enzymatic activity

Aspartate α-decarboxylase activity was determined in the crude extract of IPTG-induced *E*. *coli* BL21/RecPl and the purified recombinant *H*. *pylori* 6xHis-tagged ADC protein, according to Pei et al. (Pei et al., 2017). The reaction mixture contained either 500 μL of the crude extract and 536 μL of a 60 g/l L-aspartate solution (adjusted to pH 7.0 with NaOH) or 25 μg/mL of the purified 6xHis-tagged ADC and 1 mL of a 6.66 mg/mL L-aspartate solution (adjusted to pH 7.0 with NaOH). The assay was repeated in presence of increasing concentrations of malonic acid (0.1875, 0.375, 0.75, 1.5, 3, and 6 mg/mL equivalent to 1.8, 3.75, 7.5, 15, 30 and 60 mM). After incubating the reaction mixture at 37°C for 20 min, the reaction was stopped by adding 0.1 mL of 1 M NaOH. The production of β-alanine was measured by high performance liquid chromatography (HPLC) using Waters 2,690 Alliance HPLC system equipped with a Waters 996 photodiode array detector, Column C18 Inertsil ODS 4.6 mm × 250 mm, 5 μM Mobile phase: Acetate buffer pH 7.5: Methanol (80,20%); Mode of elution: Isocratic; Flow rate: 1 mL/min; Temperature: Ambient and Wavelength: 210 nm.

### Assessment of resistance development to ADC inhibition

*Helicobacter pylori* ATCC 43504 and *H*. *pylori* HPM001 strains were subjected to 14 consecutive serial passages in increasing malonic acid concentrations. Similarly, serial passage in increasing clarithromycin (Abbott, United States) concentrations as a comparator (Abadi, 2016) was performed according to Haas and colleagues with modifications (Haas et al., 1990). Briefly, broth microdilution testing was performed for malonic acid and clarithromycin against the tested *H*. *pylori* strains. Following incubation, a subculture from the well-containing ½ of the MIC of each drug onto Columbia agar plates supplemented with 5% sheep blood, and DENT supplement was done. The plates were incubated for 72 h at 37°C under microaerophilic conditions. At the end of incubation period, colonies (considered as P0) were suspended in saline to reach an optical density equivalent to 2.0 McFarland turbidity standard, and the process of MIC determination was repeated using P0 colonies as inoculum. The process was repeated for 14 consecutive serial passages.

### *In vitro* cytotoxicity of malonic acid

The cytotoxicity of malonic acid was assessed using sulforhodamine B (SRB) colorimetric assay against normal oral epithelial and human skin fibroblast cell lines. Cell lines were maintained in Dulbecco’s Modified Eagle Medium (DMEM); the medium for culturing cell lines was supplemented with 100 mg/mL streptomycin, 100 units/mL penicillin, and 10% FBS. Briefly, 100 μL aliquots of cell suspension (5 × 10^3^ cells) were treated in triplicates with 100 μL culture medium containing malonic acid at concentrations: 6 and 60 mg/mL and incubated for 2 h at 37°C in a humidified incubator with 5% CO_2_. Cells were fixed with 10% trichloroacetic acid and incubated at 4°C for 1 h. The trichloroacetic acid solution was removed, and the cells were washed with sterile distilled water. SRB solution (70 μL of 0.4% w/v solution) was added and incubated in a dark place at room temperature for 10 min. Plates were washed with 1% acetic acid and allowed to air-dry overnight. Tris (150 μL of 10 mM) was added to dissolve the protein-bound SRB stain, and the absorbance was measured at 540 nm using a microplate reader (Allam et al., 2018; Hosny et al., 2020). The half maximal inhibitory concentration (IC_50_) was calculated as the concentration of the test compound that inhibited the viability of the tested cells by 50% (Mahavorasirikul et al., 2010).

### *In vivo* efficiency of ADC inhibition in treatment of *Helicobacter pylori* infections

We developed a rat infection model to test the efficiency of ADC inhibition by malonic acid in treating *H*. *pylori* infections. Male Sprague–Dawley (SD) rats (10 weeks) weighing 160–200 grams, purchased from the New veterinary center (Cairo, Egypt), were used in the study (Li et al., 2018). The sample size was calculated according to the equation: 2(Z^α^_/2_ + Z^β^)^2^ × P (1–P)/(p_1_ – p_2_)^2^, where Z^α^_/2_ = Z_0.05/2_ = 1.96 (from Z table) at type 1 error of 5%; Z^β^ = Z_0.20_ = 0.842 (from Z table) at 80% power; pooled prevalence = (0.8 + 1)/2, and the value was adjusted for 5% attrition (Charan and Kantharia, 2013).

Rats were randomly assigned to Makrolon cages (3-4/cage). Environmental conditions were maintained at a temperature of 22–25°C and a 12-h light/dark cycle with food and water *ad libitum* unless fasting was required. The rats were allowed to acclimatize for 1 week prior to the experiment. All procedures involving the use of animals were performed following the recommendations of the National Institutes of Health Guide for Care and Use of Laboratory Animals (National Research Council, 2010), and were approved by the Ethics Committee of the Faculty of Pharmacy, Cairo University, Cairo, Egypt [Approval no. MI (1894)].

#### Infecting rats by *Helicobacter pylori*

Before the infection, all rats received vancomycin once daily (25 mg/kg/day), by oral gavage, for 1 week to reduce the gastric microbial load and facilitate *H*. *pylori* colonization (Stahl et al., 2014). Forty-eight rats were randomly distributed into two infection groups, each composed of 24 rats. *H*. *pylori* ATCC 43504 and *H*. *pylori* HPM001 were each used to infect the corresponding group of rats, starting from day 8 of the experiment in five infection cycles. For each infection cycle, rats were forced to fast for 18–24 h before receiving the infection dose. Three hours prior to infection, omeprazole (25 mg/kg/day), dissolved in sterile distilled water, was administered orally to reduce gastric acidity and augment the colonization process (Li et al., 1998). Infection was carried out by administering 5 × 10^8^ CFU/mL of *H*. *pylori* strain in 1 mL sterile water within 30 min of preparation, using an oro-gastric tube attached to a 3-cc or 5-cc syringe, without anaesthesia. Infection was done on days 8, 10, 12, 14, and 16 of the experiment (Werawatganon, 2014). Feeding was resumed 2 h following each infection dose (Supplementary Figure S1). An uninfected negative control group (*n* = 4) received sterile water instead of the bacterial inoculum throughout the infection process.

#### Confirmation of successful *Helicobacter pylori* infection

Successful infection of rats was confirmed using the *H*. *pylori* stool antigen (HpSA) test (ACON, United States; Asgari et al., 2020). The test was carried out according to the manufacturer’s instructions at room temperature immediately after collecting the fecal samples. The result was read within 10–20 min, where any shade of color in the test line region was considered positive. The test was performed on days 16, 23, and 30 of the experiment.

On day 30 of the experiment, we further confirmed successful infection by sacrificing three rats from each infection group and four from the negative control group. According to Li and colleagues (Li et al., 1998), with modifications, the rats were euthanized by decapitation under anesthesia using thiopental (EIPICO, Egypt). The stomach was dissected, and immediately homogenized in 20 mL brucella broth using Witeg® HG-15D homogenizer at 1500 rpm for 20 s, or until it yielded a homogenous suspension. The CLO rapid urease test (Kimberly-Clark, United States) was performed to confirm urease activity (Cai et al., 2014), following the manufacturer’s instructions, where the test result was read at room temperature within 72 h. Any change in color, from deep orange to magenta red color, was considered positive.

In addition, a sterile swab was soaked in the homogenized stomach suspension and spread in triplicates onto the surface of Columbia agar plates, supplemented with 5% sheep blood and DENT supplement. The plates were incubated for 72 h at 37°C under microaerophilic conditions. Colonies of *H*. *pylori* were identified by the characteristic morphological appearance (small translucent to pale colonies), microscopical characters (Gram negative spiral, curved or coccoid bacilli) (Andersen and Wadström, 2001), and positive oxidase (HIMEDIA, India), catalase, and urease tests (Kadkhodaei et al., 2020).

#### Malonic acid treatment

On day 38 of the experiment, each infected group (*n* = 21 rats; infected with either *H*. *pylori* ATCC 43504 or *H*. *pylori* HPM001) was subdivided into three subgroups (Supplementary Figure S2); an untreated group (*n* = 7) receiving only sterile water, group A receiving ¼ LD_50_ (327.5 mg/kg) of malonic acid once daily (*n* = 7), and group B receiving ¼ LD_50_ (327.5 mg/kg) of malonic acid twice daily (*n* = 7), by oral gavage for 3 weeks. The used LD_50_ was specified in the malonic acid manufacturer’s safety data sheet and was equivalent to 1,310 mg/kg.

Follow-up of the treatment efficiency was performed on days 7, 14, and 21 from the beginning of treatment (days 45, 52, and 59 of the experiment) using HpSA. At the end of treatment, all rats were euthanized as described earlier. The stomach was dissected, weighed, and homogenized in brucella broth. As mentioned earlier, the presence of *H*. *pylori* was determined by the CLO rapid urease test, culturing, microscopical and biochemical characteristics of the recovered isolates from stomach homogenates. The count in stomach was determined by the plate count method; the stomach’s homogenate of each rat was serially diluted (1,10, 1:100, and 1:1000), and 50 μL of each dilution was spread onto Columbia blood agar plates supplemented with 5% sheep blood, and DENT supplement and incubated for 72 h at 37°C, under microaerophilic conditions (Li et al., 1998). Following incubation, the number of *H*. *pylori* colonies was counted, and the total *H*. *pylori* count per mg stomach was calculated (Li et al., 1998).

### Assessment of the spectrum of activity of malonic acid

The spectrum of activity of malonic acid was assessed by determining malonic acid MIC against eight other pathogenic bacterial species (*A*. *baumannii* ATCC 19606, *B*. *cenocepacia* ATCC BAA-245, *E*. *coli* ATCC 25922, *E*. *faecium* ATCC 27270, *E*. *faecalis* ATCC 19433, *K*. *pneumoniae* ATCC 10031, *P*. *aeruginosa* ATCC 27856, and *S*. *aureus* ATCC 25923) using broth microdilution method, according to the CLSI guidelines (CLSI, 2018). Malonic acid solution was serially diluted in Muller-Hinton broth (100 μL) to get concentrations ranging from 6 to 0.19 mg/mL. The inoculum was prepared to be equivalent to 0.5 McFarland standard (containing approximately 1–2 × 10^8^ CFU/mL with most species) and was further diluted 1:10 (to get a concentration of 10^7^ CFU/mL). 10 μL of the diluted inoculum was transferred to each well to contain 5 × 10^5^ CFU/mL. Plates were incubated at 37°C for 20 h. The MIC was the lowest concentration that completely inhibited the visible growth of the tested organism. The experiment was carried out in triplicates.

### Statistical analysis

All statistical analyses were performed using GraphPad Prism 8.0.1. Two-way ANOVA and the unpaired t-test were used to evaluate the results of determining the MIC of malonic acid. Two-way ANOVA and multiple t tests, using the Holm-Sidak method, were used to analyse the results of *in vitro* confirmation of ADC inhibition by malonic acid, treatment follow-up using HpSA test, and the results of the *H*. *pylori* total plate count. The difference was significant at *p* < 0.05 in all tests. *Post hoc* test using Dunnett’s method was used to evaluate the significance of the results of *H*. *pylori* total plate count in treated and untreated groups. Pearson correlation coefficient was used to evaluate the correlation between different concentrations of β-alanine and pantothenate and the MIC of malonic acid as well as the correlation between different malonic acid concentrations and ADC enzymatic activity.

## Results

### Successful docking of malonic acid to ADC binding site

**Figure 1:**
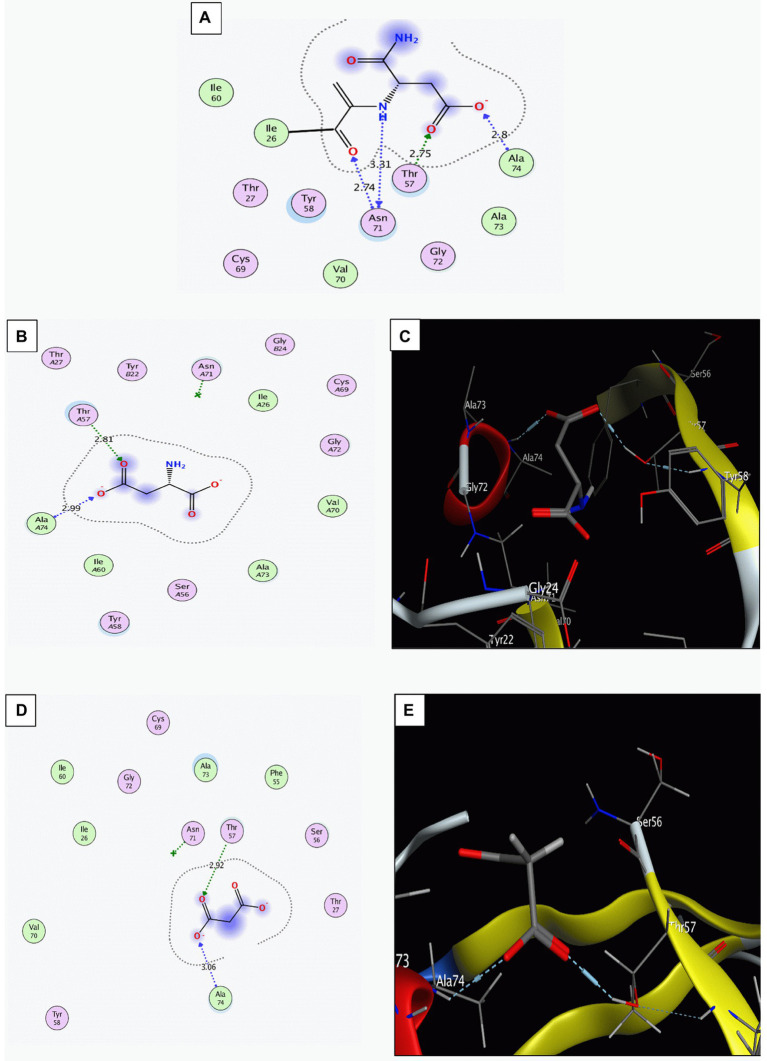
Interaction of the binding site of aspartate α-decarboxylase with different ligands. **(A)** The co-crystalized ligand ((N-2-(2-amino-1-methyl-2-oxoethylidene)asparaginate)). **(B,C)** Aspartate (Two-D structures and three-D structures). **(D,E)** Malonate (Two-D structures and three-D structures). Images were generated by Molecular Operating Environment (MOE, 2015.10) software.

Multiple sequence alignment of the sequences of ADC enzyme from *H*. *pylori* 26695 (PDB ID: 1UHE), *H*. *Pylori* ATCC 43504, and from other randomly selected *H*. *Pylori* strains showed high level of conservation (Supplementary Figure S3). Malonic acid interaction with the ADC binding site was modeled *via* molecular docking. The docking setup was first validated by self-docking the co-crystallized potential ligand [N-2-(2-amino-1-methyl-2-oxoethylidene) asparaginate] in the vicinity of the binding site of the ADC. It bound the amino acids: Isoleucine (Ile) 26, Threonine (Thr) 57, Asparagine (Asp) 71, and Alanine (Ala) 74 through ionic, hydrogen bond, and hydrophobic interactions, with a docking score of −4.4312 kcal/mol (Figure 1A). Docking of aspartate (ADC substrate) with ADC was used to identify its intermolecular interactions with the active binding site. Aspartate interacted with the amino acids Thr57 and Ala74 through hydrogen bonding and hydrophobic interactions, with a docking score of −3.9130 kcal/mol (Figures 1B,C). The validated setup was then used to predict the ligand-receptor interactions for malonate with the ADC binding site. Malonate interacted with the key amino acids in the binding site (Thr57 and Ala74) through hydrogen bonding and hydrophobic interactions, with a docking score of −3.5542 kcal/mol (Figures 1D,E).

### *In vitro* anti-helicobacter activity of malonic acid

The MIC of malonic acid was determined by agar dilution and broth microdilution methods against *H*. *pylori* ATCC 43504 and *H*. *pylori* HPM001. The MIC against both strains was 0.75 mg/mL when using the agar dilution method. Upon using the broth microdilution method, the MIC was 0.5 ± 0.17 and 0.75 mg/mL against *H*. *pylori* ATCC 43504 and *H*. *pylori* HPM001. The MBC of malonic acid was 1.5 mg/mL for both strains. No significant difference was recorded between the MIC of malonic acid against *H*. *pylori* ATCC 43504 and *H*. *pylori* HPM001 using either the agar (*p* = 0.8529) or the broth microdilution methods (*p* = 0.8784). There was also no significant difference between the mean MIC of malonic acid recorded against the tested strains by both methods (*p* = 0.54).

### ADC inhibition by malonic acid

**Figure 2:**
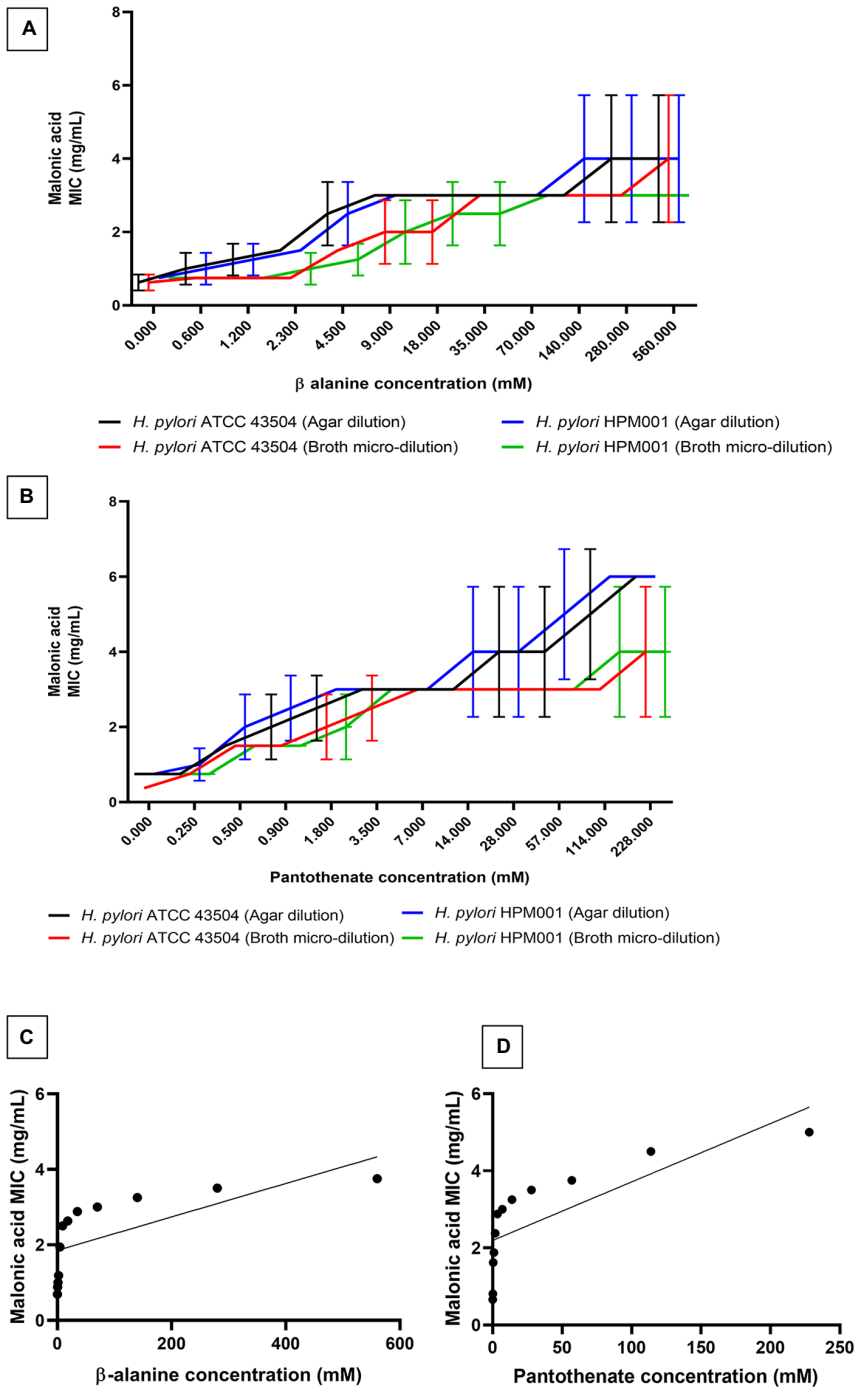
Minimum inhibitory concentration (MIC) of malonic acid in presence of the products of aspartate α-decarboxylase catalyzed enzymatic reaction. MIC of malonic acid in the presence of increasing concentrations of **(A)** β-alanine and **(B)** pantothenate, using both agar dilution and broth microdilution methods, against both *Helicobacter pylori* ATCC 43504 and the clinical *H*. *pylori* isolate (HPM001). The correlation between the MIC of malonic acid and the concentrations of **(C)** β-alanine, and **(D)** pantothenate.

Confirmation of ADC inhibition by malonic acid was carried out by determining the MIC of malonic acid in the presence of increasing sub-inhibitory concentrations of β-alanine and pantothenate (the products of the inhibited enzymatic reaction). The MIC of β-alanine and pantothenate were 1.12 M (100 mg/mL) and 456 mM (100 mg/mL), respectively. Supplementing the growth media with increasing concentrations of β-alanine or pantothenate resulted in a significant increase in the mean malonic acid MIC (Figures 2A,B) determined using the agar dilution and broth microdilution methods. A strong uphill linear relationship between β-alanine (*r* = 0.6857) or pantothenate (*r* = 0.7670) concentrations and the MIC of malonic acid was observed (Figures 2C,D).

**Figure 3:**
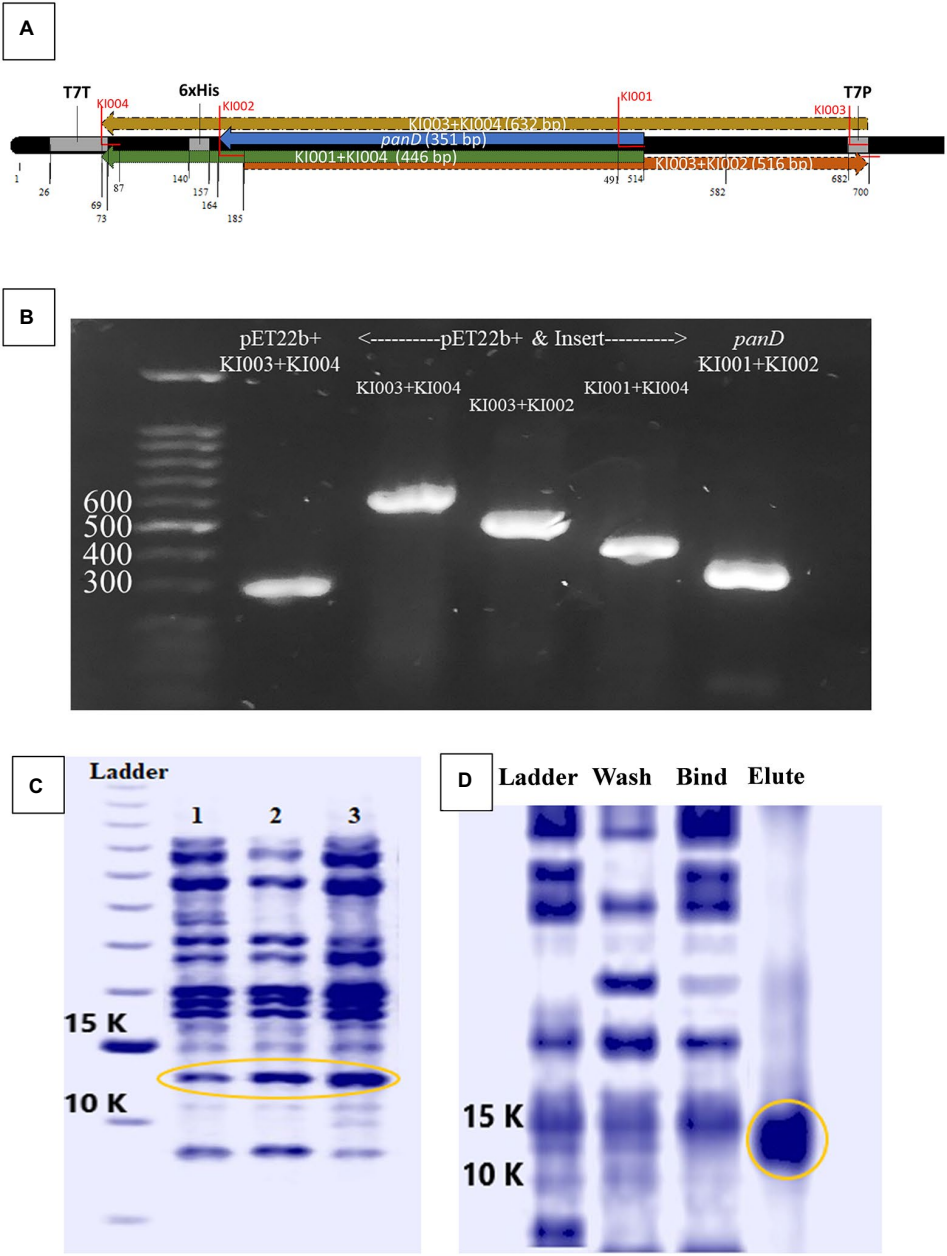
Cloning and expression of *Helicobacter pylori* ATCC 43504 aspartate α-decarboxylase. **(A)** Schematic diagram generated by BioEdit (7.2.5., 2015) of pET22b + vector containing the *pan*D insert with the positions of different primers highlighted. **(B)** Polymerase chain reaction (PCR) amplicons produced by different primer combinations performed on *H*. *pylori* ATCC 43504 DNA (*pan*D), empty pET22b(+) plasmid vector and the recombinant plasmid vector pET22b(+) containing the insert *pan*D, **(C)** Crude protein extract from: lane 1: *Escherichia coli* BL21, lane 2: *E*.*coli* BL21/RecPl induced by 0.5 mM Isopropyl β-D-1-thiogalactopyranoside (IPTG), lane 3: *E*. *coli* BL21/RecPl induced by 0.75 mM IPTG **(D)** The wash, bind and elute of Ni-NTA columns purification of the recombinant protein from *E*. *coli* BL21/RecPl.

**Figure 4:**
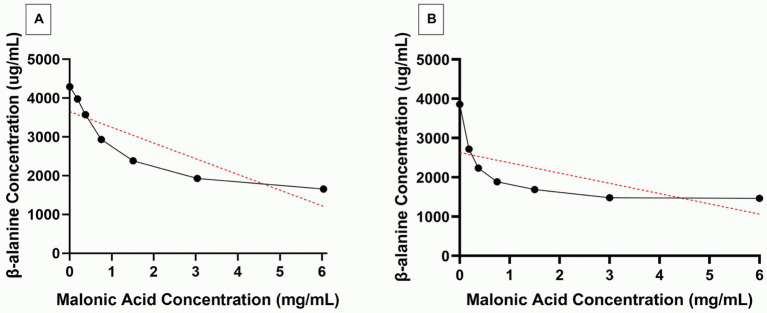
Selective inhibition of *Helicobacter pylori* aspartate α-decarboxylase (ADC) by malonic acid. β-alanine (Black line) produced from the action of **(A)** crude enzyme extract and **(B)** purified 6x-His tagged ADC, of Isopropyl β-D-1-thiogalactopyranoside induced *Escherichia coli* BL21/RecPl, in the presence of increasing concentrations of malonic acid. The red lines depict the correlation between the two variables.

Further confirmation of ADC inhibition by malonic acid was carried out by assaying β-alanine, the product of the enzymatic action of recombinant *H*. *pylori* 6x-His-tagged ADC on aspartate, in the presence of an increasing concentration of malonic acid using HPLC. The *pan*D gene, encoding ADC, was amplified from *H*. *pylori* ATCC 43504 genome, resulting in a DNA fragment of 375 bp; the DNA fragment was inserted in the pET22b + plasmid. The constructed recombinant RecP1 plasmid (pET22b + with *panD* insert) was transformed into *E*. *coli* DH5α. Clones were confirmed by PCR using different combinations of primers (Figures 3A,B). The cloned DNA fragment encoded 123 amino acids polypeptide, 117 amino acids for ADC, and six amino acids for the His-tag. The whole fragment was predicted to have a size of 13.8 kDa using (Bioinformatics.Org/sms/prot_mw.html). The recombinant plasmid RecP1 was transformed into *E*. *coli* BL21, and the recombinant protein was purified using Ni-NTA columns. A single intense band (37% of the elute) with the expected size of 13.8 KDa was visualized on SDS-PAGE (Figures 3C,D). A standard curve for β-alanine was constructed using HPLC (Supplementary Figure S4). The ADC activity of the crude extract of IPTG-induced *E*. *coli* BL21/RecPl and the purified 6x-his tagged ADC was measured. There was a decrease in the concentration of the produced β-alanine by increasing malonic acid concentration. A strong downhill linear relationship between β-alanine and malonic acid concentrations was recorded in crude and purified enzyme preparations (*r* = −0.8605 and − 0.6575, respectively; Figure 4).

### Lack of resistance development in *Helicobacter pylori* by repeated ADC inhibition using malonic acid

**Figure 5:**
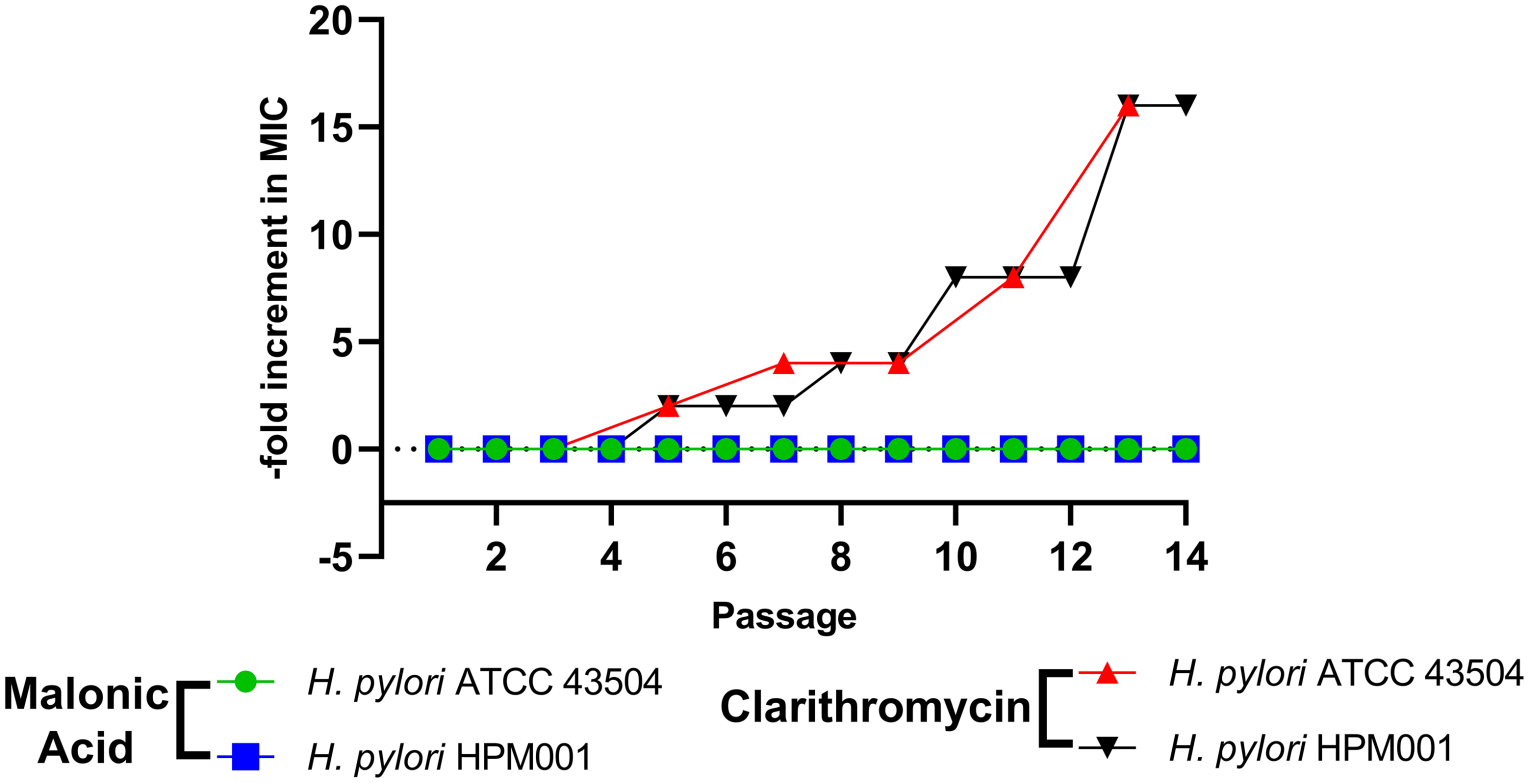
Lack of resistance development in *Helicobacter pylori* by repeated exposure to aspartate α-decarboxylase inhibition using malonic acid. Fold increase in minimum inhibitory concentration (MIC) of malonic acid and clarithromycin against *H*. *pylori* ATCC 43504 and the clinical *H*. *pylori* HPM001 isolate, following 14 serial passages.

*Helicobacter pylori* ATCC 43504 and *H*. *pylori* HPM001 were subjected to serial passage in the presence of increasing concentrations of either malonic acid or clarithromycin (comparator). No increase in the MIC of malonic acid against both strains was recorded after 14 serial passages. However, there was an increase in the MIC of clarithromycin against both strains. The MIC of clarithromycin against *H*. *pylori* ATCC 43504 and *H*. *pylori* HPM001 was 6 and 48 μg/mL and increased by 16-fold, following 14 serial passages (Figure 5).

### Malonic acid is not cytotoxic

The *in vitro* cytotoxicity of malonic acid was evaluated in oral epithelial and human skin fibroblast cell lines using two concentrations, 6 mg/mL (equivalent to 10X the average MIC) and 60 mg/mL (approximately the *in vivo* used dose per rat). The IC_50_ was higher than 60 mg/mL, with recorded cell viability of 82.13% ± 2.13 and 61.36% ± 2.25 in oral epithelial cells and 85.91 ± 1.1% and 79.03 ± 0.94% in human skin fibroblast for 6 and 60 mg/mL, respectively.

### Successful treatment of *Helicobacter pylori* infection by ADC inhibition

*Helicobacter pylori* ATCC 43504 and *H*. *pylori* HPM001were used to infect SD male rats (*n* = 48). An uninfected group that received only sterile water served as a negative control (*n* = 4). The HpSA test was used to monitor infection; on day 16 (the day of the last infection cycle), all rats (*n* = 52) tested negative. The following week (day 23), 87.5% (*n* = 21) and 83.3% (*n* = 20) of *H*. *pylori* ATCC 43504 and *H*. *pylori* HPM001 infected rats tested positive, and by day 30, all infected rats tested positive. Rats in the uninfected control group remained negative throughout the experiment.

Successful infection was further confirmed by sacrificing three rats from each infected group, besides the four uninfected rats, to validate the HpSA test results. The rapid urease CLO test was performed on the homogenized stomachs of the sacrificed rats. Homogenized stomachs of all infected rats tested positive for urease, while those of uninfected rats tested negative. Colonies resulting from culturing the homogenized stomachs of infected rats were positive for urease, oxidase, and catalase. Microscopically examining the Gram-stained colonies under 1,000× magnification revealed the characteristic spiral-shaped Gram-negative single rods indicative of *H*. *pylori*. No growth was observed when culturing the stomachs of uninfected rats.

**Figure 6:**
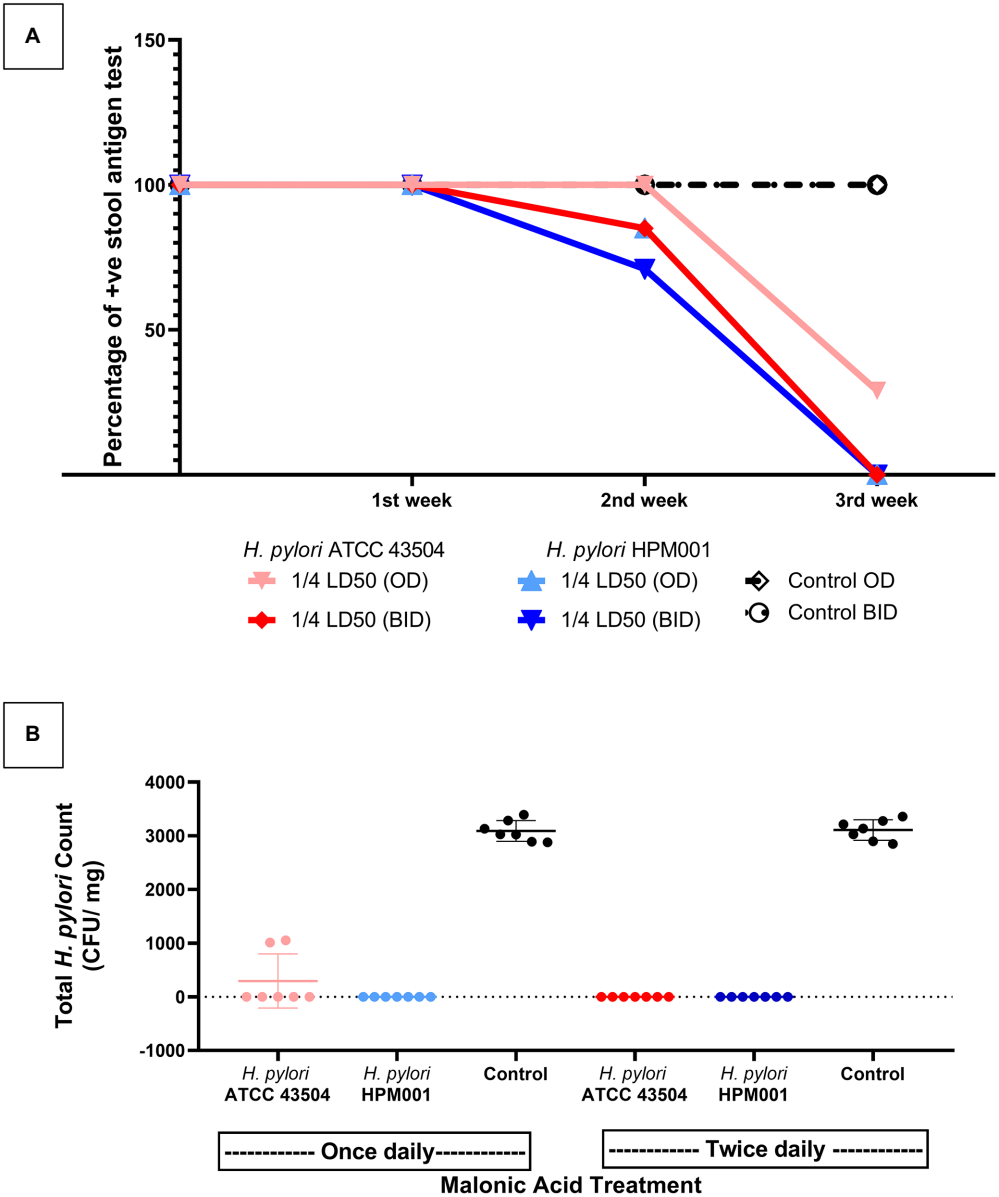
Inhibition of aspartate-α-decarboxylase successfully treated *Helicobacter pylori* infected rats. **(A)** Weekly follow up of the number of rats that cleared infection with either *H*. *pylori* ATCC 43504 or the clinical *H*. *pylori* HPM001 strains as indicated by *H*. *pylori* Stool Antigen (HpSA) test during the 3 weeks treatment period. **(B)** The total *H*. *pylori* count in the stomachs of malonic acid treated and untreated (control) groups of rats at the end of the 3 weeks treatment period. OD, once daily; BID, twice daily; LD_50_, lethal dose 50; Control, rat groups that received sterile water instead of malonic acid.

Infected rats with *H*. *pylori* ATCC 43505 and *H*. *pylori* HPM001 (*n* = 21 each) were treated with malonic acid (¼ LD_50_ once (*n* = 7) and twice (*n* = 7) daily); in addition to two control untreated groups (*n* = 7 each) that received sterile water once and twice daily (Supplementary Figure S5). All rats survived the entire treatment period. The untreated rats (*n* = 14) tested positive for *H*. *pylori* by the HpSA test throughout the 3-weeks treatment period. All treated rats (*n* = 42) tested positive for *H*. *pylori* after the first week of treatment. The number of rats that tested negative in the HpSA test increased by the end of the second week of treatment to reach 15% (*n* = 4). This includes three rats treated with ¼ LD_50_ twice daily and one treated with ¼ LD_50_ once daily. By the end of the 3 weeks treatment period, 93% of the infected rats in the treatment groups (*n* = 26) tested negative in the HpSA test. Only two rats failed to clear the infection; they were treated with ¼ LD_50_ (*n* = 2) once daily (Supplementary Figure S5). Follow-up of the number of rats clearing the infection following each week of treatment is shown in Figure 6A.

By the end of the treatment period, all rats (*n* = 42) were sacrificed, and the stomachs were dissected, weighed, and homogenized. Performing the rapid urease CLO test on homogenized stomachs confirmed the HpSA test results. Upon culturing the homogenized stomachs, no visible growth was observed in rats testing negative with the HpSA test; the total *H*. *pylori* plate count of the untreated group (*n* = 14) ranged from 2,850 to 3,400 CFU/mg stomach. However, the homogenized stomachs of the two rats that failed to clear the infection (treated with ¼ LD_50_ once daily and tested positive with the HpSA test at the end of the treatment period) had a mean total *H*. *pylori* plate count of 1,030 CFU/mg stomach. There is a significant difference between the mean of the total *H*. *pylori* counts in the stomachs of rats that failed to clear the infection (treated with ¼ LD_50_ of malonic acid once daily) and the mean of the total *H*. *pylori* counts in the stomachs of the untreated group (*p* < 0.0001; Figure 6B).

### The broad-spectrum of MA as an ADC inhibitor

Malonic acid MIC was determined against other pathogenic species (*A*. *baumannii* ATCC 19606, *B*. *cenocepacia* ATCC BAA-245, *E*. *coli* ATCC 25922, *E*. *faecium* ATCC 27270, *E*. *faecalis* ATCC 19433, *K*. *pneumoniae* ATCC 10031, *P*. *aeruginosa* ATCC 27856, and *S*. *aureus* ATCC 25923) by the broth microdilution method. The MIC of malonic acid ranged from 0.625 to 1.25 mg/mL in the tested species (Supplementary Table S2).

## Discussion

Aspartate α-decarboxylase was previously reported, using *in silico* proteomic approaches, as a promising conserved drug target in *H*. *pylori* with malonic acid as the proposed inhibitor (Ibrahim et al., 2020). Here, the conservation of ADC in 50 non-redundant *H*. *pylori* strains, including the strain for which the crystallographic structure of ADC enzyme was available in the protein databank (*H*. *pylori* 26695) and the standard *H*. *pylori* ATCC 43504 strain, was confirmed. We modeled the interaction of malonic acid to *H*. *pylori* ADC *via* molecular docking, which resulted in a docking score (−3.5542) similar to that of its natural substrate, aspartate (−3.9130). Both interacted with the key amino acids in the ADC binding site using similar bonds (hydrogen bonding and hydrophobic interactions); these key amino acids (Thr57 and Ala74) are totally conserved in the aligned ADC sequences. Aspartic acid (2-amino-butanedioic acid, C_4_H_7_NO_4_) and malonic acid (propanedioic acid, C_3_H_4_O_4_) are structurally similar, with the latter being one carbon less. A low docking score with good interaction results reveals the stability of the ligands and receptors interactions (Syahri et al., 2017). Interaction of the inhibitor with weak bonds to the enzyme’s binding site suggests reversible competitive inhibition (Kwon et al., 2022). ADC enzyme was reported as a drug target in *M*. *tuberculosis* and could be inhibited by pyrazinamide (Gopal et al., 2020).

The MIC of malonic acid against *H*. *pylori* ranged from 0.5 to 0.75 mg/mL. This is the first report about the anti-helicobacter activity of malonic acid. Few studies have reported the antibacterial activity of malonic acid as a component of the pine needles extract (Feng et al., 2010), and in ternary complexes (El-Sherif, 2010). Several studies reported using short-chain acids as antimicrobial agents (Dittoe et al., 2018; El Baaboua et al., 2018; Gómez-García et al., 2019; Kovanda et al., 2019), besides the prolonged use of organic acids as food preservatives (Ricke, 2003). Malonic acid and its salts are known inhibitors of succinate dehydrogenase enzyme, involved in the cellular respiration as a part of Kreb’s cycle, of different bacterial species including *H*. *pylori* (Chen et al., 1999; Minato et al., 2013; Meylan et al., 2017; Matsumoto et al., 2020). Inhibition of succinate dehydrogenase was reported to be responsible for the antimicrobial activity of many natural compounds (Keohane et al., 2018; Guo et al., 2021). However, in our previous *in silico* study on druggable targets in *H*. *pylori*, succinate dehydrogenase was not retrieved among the list of essential or choke points proteins of *H*. *pylori* (Ibrahim et al., 2020).

The results of the agar dilution and broth microdilution methods, used for MIC determination, were comparable, with no significant difference. This agrees with other studies comparing both methods against *H*. *pylori* (Piccolomini et al., 1997), and *H*. *cinaedi* (Tomida et al., 2013). The agar dilution method, the CLSI approved method for antimicrobial susceptibility testing (CLSI, 2015), is time-consuming and tedious compared to the broth microdilution method. Therefore, the broth microdilution method can be an alternative to the agar dilution method for determining MIC in *H*. *pylori*. Moreover, the difference in the MIC value between *H*. *pylori* ATCC 43504 and the clinical *H*. *pylori* HPM001 strain, determined by both methods, was non-significant.

The MBC of malonic acid was almost only two-fold its MIC, confirming the bactericidal nature of ADC inhibition in *H*. *pylori* by malonic acid. Antimicrobial agents are bactericidal if the MBC is not more than four-fold the MIC (French, 2006).

The selective ADC inhibition by malonic acid was further confirmed by the significant dose-dependent increase in malonic acid MIC (*p* < 0.05) in presence of increasing sub-inhibitory concentrations of β-alanine and pantothenate (the end products of the enzymatic reaction catalyzed by ADC enzyme), with a strong uphill positive relationship between either β-alanine (*r* = 0.6857) or pantothenate (*r* = 0.767) concentrations and malonic acid MIC. Similarly, pyrazinamide, the first-line anti-tuberculosis agent, interferes with CoA biosynthesis in *M*. *tuberculosis* by inhibiting the ADC enzyme (Shi et al., 2014; Gopal et al., 2020). β-alanine and pantothenate also antagonize the activity of pyrazinamide in *M*. *tuberculosis* (Shi et al., 2014). Pantothenate auxotrophic strains of *M*. *tuberculosis* are insensitive to pyrazinamide, while prototrophic strains are sensitive (Dillon et al., 2014). Similarly, *Zymomonas mobilis* pantothenate auxotrophs grow well in media supplemented with β-alanine (Gliessman et al., 2017). Several natural and synthetic pantothenic acid analogues possess anti-bacterial activity (Spry et al., 2008). Humans depend on the exogenous uptake of pantothenic acid, while some bacteria, plants, and fungi are capable of *de novo* synthesis of pantothenic acid from β-alanine (Webb et al., 2004). The absence of ADC enzyme in humans makes it a specific promising drug target (Sharma et al., 2012).

The activity of the recombinant 6x-His-tagged ADC enzyme was assayed in presence of increasing concentrations of malonic acid to further confirm ADC inhibition by malonic acid. Direct measurement of the β-alanine concentration, by HPLC, in the enzymatic reaction catalyzed by either the crude extract of IPTG-induced *E*. *coli* BL21/RecPl or the purified recombinant 6x-His-tagged ADC showed a significant reduction in enzymatic activity with increasing malonic acid concentration (*p* < 0.000001).

Repeated exposure to ADC inhibition by malonic acid did not develop resistance in any of the tested *H*. *pylori* strains. In contrast, clarithromycin-resistant and susceptible *H*. *pylori* strains readily developed resistance following 14 serial passages, with the MIC increasing by 16-fold at the end of the passages. Similar studies reported an increased MIC of clarithromycin against *H*. *pylori* isolates following repeated exposure (Kobayashi et al., 2002). This confirms the superiority of ADC inhibition as a drug target in *H*. *pylori*, where the relapse of *H*. *pylori* infection usually occurs due to incomplete eradication or the emergence of resistant strains (Abadi, 2016).

Malonic acid had no cytotoxic effect on the tested oral epithelial or human skin fibroblast cells, even at the highest tested concentration (60 mg/mL). According to the US national cancer institute guidelines, any compound is considered to lack cytotoxic activity if it has an IC_50_ > 4 μg/mL (Kroll, 2001; Ramasamy et al., 2012).

The effectiveness of ADC inhibition in treating *H*. *pylori* infection was tested in a SD rat infection model. Developing a successful *H*. *pylori* infection model is challenging as the infection models take a long time with high failure rates (Taylor and Fox, 2012; Werawatganon, 2014). Infection with *H*. *pylori* Sydney strain remains the most successful animal model for *H*. *pylori* infection (Taylor and Fox, 2012). However, infection with other strains like *H*. *pylori* B128 and *H*. *pylori* ATCC 43504 was also successful (Israel et al., 2001; Hahm et al., 2002; Fox et al., 2003). Non-toxigenic *H*. *pylori* strains often fail to induce successful animal infection compared to the *cag*A-positive and *vac*A-positive strains (Werawatganon, 2014). We used *H*. *pylori* ATCC 43504 and a clinical *cag*A-positive and *vac*A-positive *H*. *pylori* isolate (*H*. *pylori* HPM001) to infect SD male rats.

Our model was successful, as *H*. *pylori* colonized ~85.4% of rats by the end of first week post-infection and 100% by the end of the second week. The HpSA test was used to monitor infection throughout the study; it has been used previously to detect *H*. *pylori* infection in C57BL/6 mice and the results were validated by PCR and rapid urease test (Sjunnesson et al., 2003; Moon et al., 2013). We validated the HpSA test results using the rapid urease test and culture techniques. The results of oxidase, catalase, and urease tests performed on colonies from cultured stomachs of infected and non-infected rats matched the HpSA test.

Aspartate α-decarboxylase Inhibition by malonic acid was effective in the complete eradication of *H*. *pylori* infection when ¼ LD_50_ (327.5 mg/kg) of malonic acid was administered twice daily for 3 weeks. This dosing regimen was optimum in terms of safety and effectiveness against *H*. *pylori*. Administering malonic acid at ¼ LD_50_ (327.5 mg/ kg) once daily for 3 weeks resulted in a 100% survival rate and 85.7% curing following the 3 weeks treatment period. However, the average *H*. *pylori* plate count of the homogenized stomachs from the non-cured rats showed a significant difference (*p* < 0.0001) from the average *H*. *pylori* plate count of untreated infected rats. Prolonging the treatment period with the once-daily dose regimen could cause complete curing. This is evidenced by the significant difference (*p* < 0.0001) in the percentage of the HpSA positive results recorded in the first and second week of treatment and that recorded in the second and third week of treatment. The percentage of positive HpSA test results following each week of treatment declined slowly in the group receiving ¼ LD_50_ (327.5 mg/ kg) once daily (mean positive results following each week of treatment were 100, 92.8, and 14.2%, respectively). However, this decline was moderate and steady in the group treated with ¼ LD_50_ (327.5 mg/ kg) twice daily (mean positive test results following each week of treatment were 100, 78.6, and 0%, respectively). This agrees with previous studies about the significant impact of prolonging treatment, either from seven to 10 days or from 10 to 14 days, on eradicating *H*. *pylori* infection in man (Lee et al., 2010; Fallone et al., 2013; Yuan et al., 2017).

The inhibition of succinate dehydrogenase by malonate in different models (mice and rats) is known to modulate tissue inflammation (Yang et al., 2019; Jespersen et al., 2020); however, whether the use of malonate derivatives in treatment of *H*. *pylori* infection will also result in modulating gastric tissue inflammation needs to be tested.

Aspartate α-decarboxylase was confirmed as a broad-spectrum target, with comparable malonic acid MIC, in eight bacterial species other than *H*. *pylori*. *P*. *aeruginosa* and *E*. *faecalis* had the lowest MIC (0.625 mg/mL), similar to the mean MIC recorded against *H*. *pylori* (0.6875 mg/mL). This agrees with our previous *in silico* results regarding the possible broad-spectrum of ADC as a drug target (Ibrahim et al., 2020).

The high MIC values recorded with malonic acid will hinder its applicability in treatment of patients with *H*. *pylori* infections. Nevertheless, we present malonic acid as a non-toxic lead molecule that can be structurally modified to produce an effective anti-helicobacter agent. Future studies will then be required to determine the kinetics of enzyme inhibition of the newly-developed inhibitors and whether these inhibitors will have an inhibitory effect on succinate dehydrogenase.

The successful use of *in silico* approach in prediction of novel therapeutic targets in microbial species was described previously (Kaplan et al., 2012; Serral et al., 2021). This study is another example of using *in silico* approach in predicting druggable targets in pathogenic species and their possible ligands that can be utilized as lead molecule for the development of novel antimicrobial agents.

## Conclusion

Aspartate α-decarboxylase is a promising drug target in *H*. *pylori*, with low tendency for resistance development by repeated exposure. Malonic acid can be a lead molecule for developing effective anti-helicobacter compounds functioning through ADC inhibition. This offers new hope for saving the lives of those at high risk of infection with the carcinogenic *H*. *pylori* pathogen.

The determination of *H*. *pylori* MIC by broth microdilution method is comparable to the gold standard agar dilution method. The broth dilution method is much easier to perform and more efficient in terms of cost and time. Additionally, we successfully developed *H*. *pylori* infection model by strains other than the Sydney strain in SD rats that can be used for further *in vivo* testing.

## Data availability statement

The original contributions presented in the study are included in the article/supplementary material, further inquiries can be directed to the corresponding author.

## Ethics statement

The animal study was reviewed and approved by Ethics Committee of the Faculty of Pharmacy, Cairo University, Cairo, Egypt [Approval no. MI (1894)].

## Author contributions

MK, MR, and OH contributed to the study conception and design. KI performed the experiment and wrote the first draft of the manuscript. KI, MK, and OH contributed to data analysis and provided the required resources for the work. MK, TE, MR, and OH supervised the work. MK and OH contributed in the preparation of the final article. All authors approved the submitted version.

## Funding

Publication fees are partially covered by Cairo University.

## Conflict of interest

The authors declare that the research was conducted in the absence of any commercial or financial relationships that could be construed as a potential conflict of interest.

## Publisher’s note

All claims expressed in this article are solely those of the authors and do not necessarily represent those of their affiliated organizations, or those of the publisher, the editors and the reviewers. Any product that may be evaluated in this article, or claim that may be made by its manufacturer, is not guaranteed or endorsed by the publisher.
