# Supplementary material for: Aspartate α-decarboxylase a new therapeutic target in the fight against Helicobacter pylori infection
Source: Front Microbiol. 2022 Nov 7;13:1019666. doi: 10.3389/fmicb.2022.1019666 (PMC9746714; doi:10.3389/fmicb.2022.1019666)
Supplement: Supplementary file 1 [file Table_1.DOCX]

**Supplementary Table 1.** **List of primers used in the study**

| **Primer** | **Sequence (5ʹ-3ʹ)** | **Description** |
| --- | --- | --- |
| KI001 | AGGTGAGAATTCATGACTTTTGAAATGCTTTATAGT | Binds to the *H. pylori* ATCC 43504 genome (Genbank accession number AP017632.1) at nucleotide number 242138. The recognition sequence of the *Eco*RI restriction enzyme is underlined. |
| KI002 | AATCCACTCGAGACCCTTTTCTAAAATTTCGTT | Binds to the *H. pylori* ATCC 43504 genome (Genbank accession number AP017632.1) at nucleotide number 242491. The recognition sequence of the *Xho*I restriction enzyme is underlined. |
| KI003 | TAATACGACTCACTATAGGG | Binds to the T7 promotor of the pET-22b(+) plasmid at nucleotide number 377 on the antisense strand (Yang et al., 2020) |
| KI004 | GCTAGTTATTGCTCAGCGG | Binds to the T7 terminator of the pET-22b(+) plasmid at nucleotide number 69 on the sense strand (Yang et al., 2020) |
