# Supplementary material for: Aspartate α-decarboxylase a new therapeutic target in the fight against Helicobacter pylori infection
Source: Front Microbiol. 2022 Nov 7;13:1019666. doi: 10.3389/fmicb.2022.1019666 (PMC9746714; doi:10.3389/fmicb.2022.1019666)
Supplement: Supplementary file 2 [file Table_2.DOCX]

**Supplementary Table 2.** **The mean minimum inhibitory concentrations of malonic acid against different bacterial species**

| **Bacterial species** | **Mean MIC (mg/mL)** |
| --- | --- |
| *Helicobacter pylori* ATCC 43504 | 0.625 ± 0.18 |
| *Helicobacter pylori* HPM001 | 0.75 ± 0.00 |
| *Acinetobacter baumannii* ATCC 19606 | 0.75 ± 0.00 |
| *Burkholderia cepacia* ATCC BAA-245 | 1 ± 0.35 |
| *Enterococcus faecalis* ATCC 19433 | 0.625 ± 0.18 |
| *Enterococcus faecium* ATCC 27270 | 1 ± 0.35 |
| *Escherichia coli* ATCC 25922 | 0.75 ± 0.00 |
| *Klebsiella pneumoniae* ATCC 10031 | 1.25 ± 0.35 |
| *Pseudomonas aeruginosa* ATCC 27856 | 0.625 ± 0.18 |
| *Staphylococcus aureus* ATCC 25923 | 0.75 ± 0.00 |
