# Supplementary material for: Aspartate α-decarboxylase a new therapeutic target in the fight against Helicobacter pylori infection
Source: Front Microbiol. 2022 Nov 7;13:1019666. doi: 10.3389/fmicb.2022.1019666 (PMC9746714; doi:10.3389/fmicb.2022.1019666)
Supplement: Supplementary file 3 [file Data_Sheet_1.docx]

Supplementary Material

# Supplementary Figures


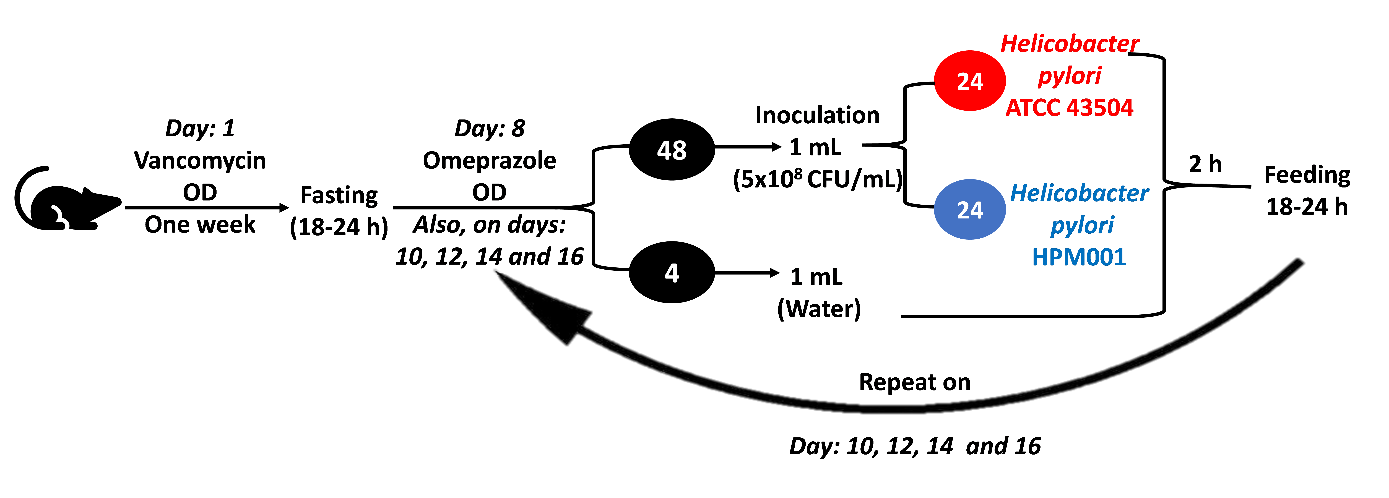


**Supplementary Figure 1.** **Induction of infection with *H. pylori* in Sprague- Dawley rats*.***

A schematic diagram of the *H. pylori* infection model developed in the Sprague-Dawley rats

**OD:** Once daily


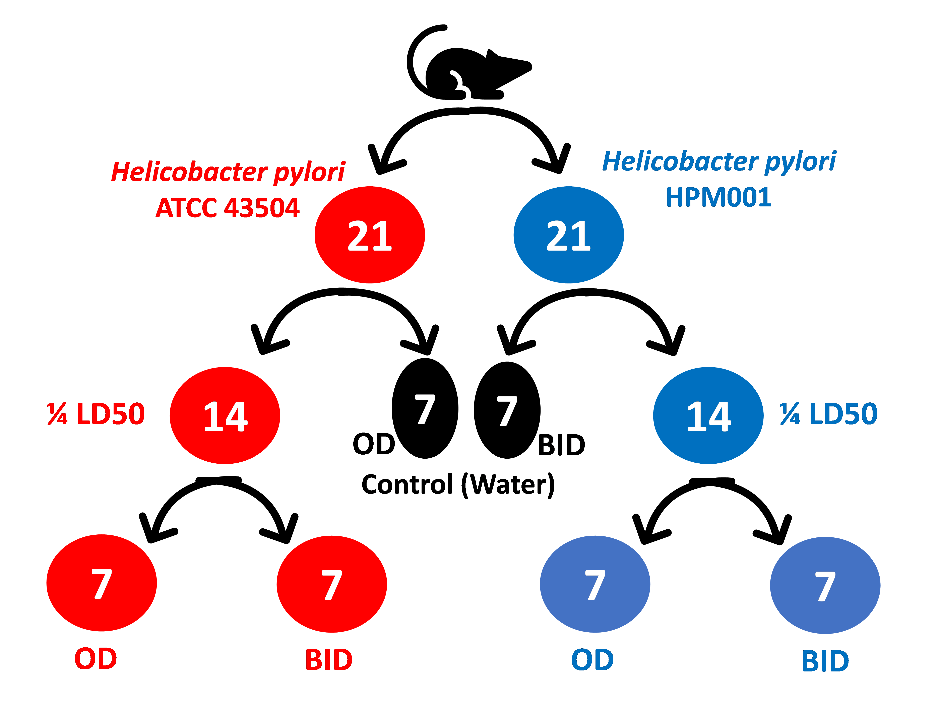


**Supplementary Figure 2.** **The treatment regimens for *H. pylori* infected rats.**

A schematic diagram of malonic acid treatment regimens for *H. pylori* infected rats

**LD_50_:** Lethal dose 50; **OD:** Once daily; **BID:** Twice daily


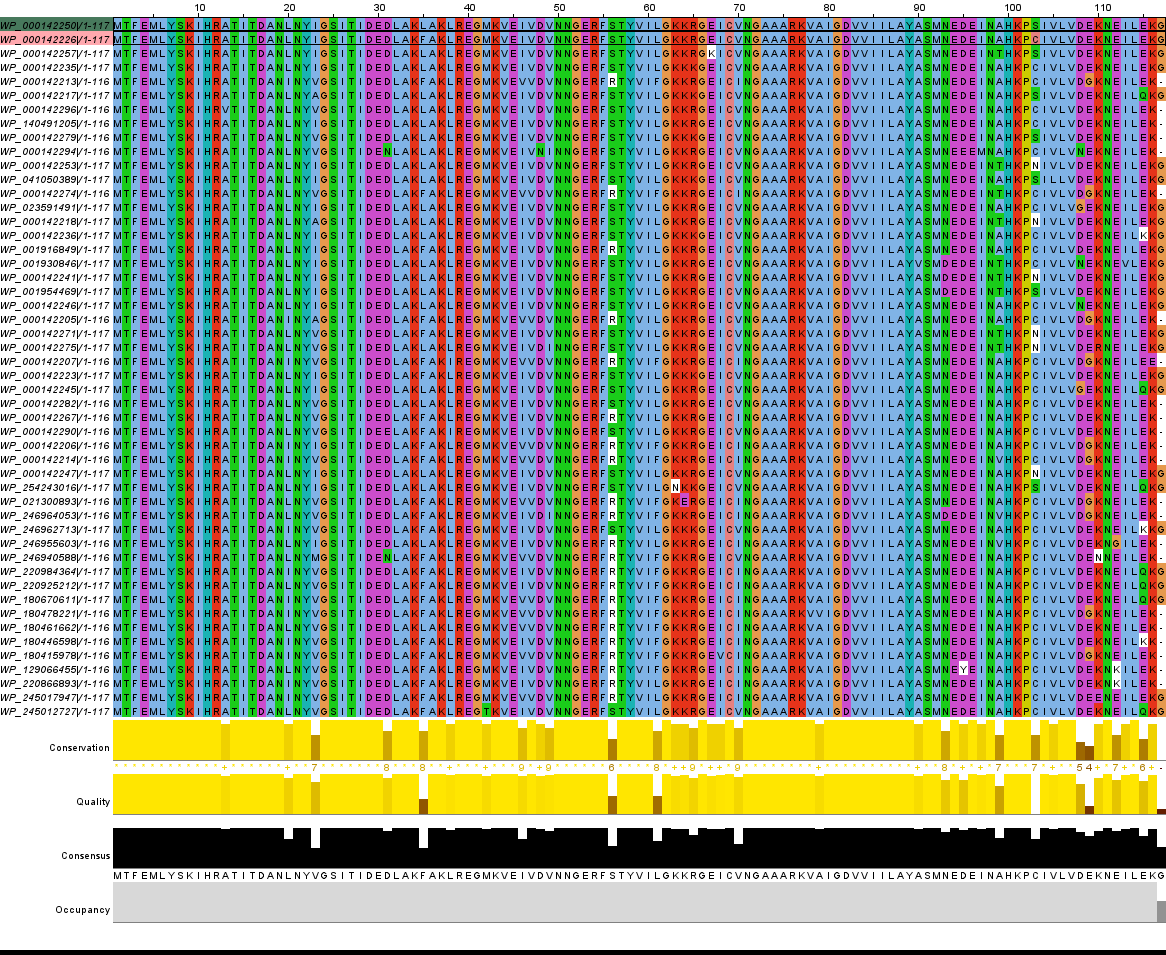


**Supplementary Figure 3. Conservation of aspartate α-decarboxylase in different *Helicobacter pylori* strains.**

Alignment of the amino acid sequences of aspartate α-decarboxylase enzyme in non-redundant *H. pylori* strains, generated by Clustal Omega. *H. pylori* ATCC 43504 is highlighted by pink color and *H. pylori* 26695 is highlighted by green color. The degree of conservation of each amino acid is indicated by the yellow columns below the figure where those with asterisk are totally conserved


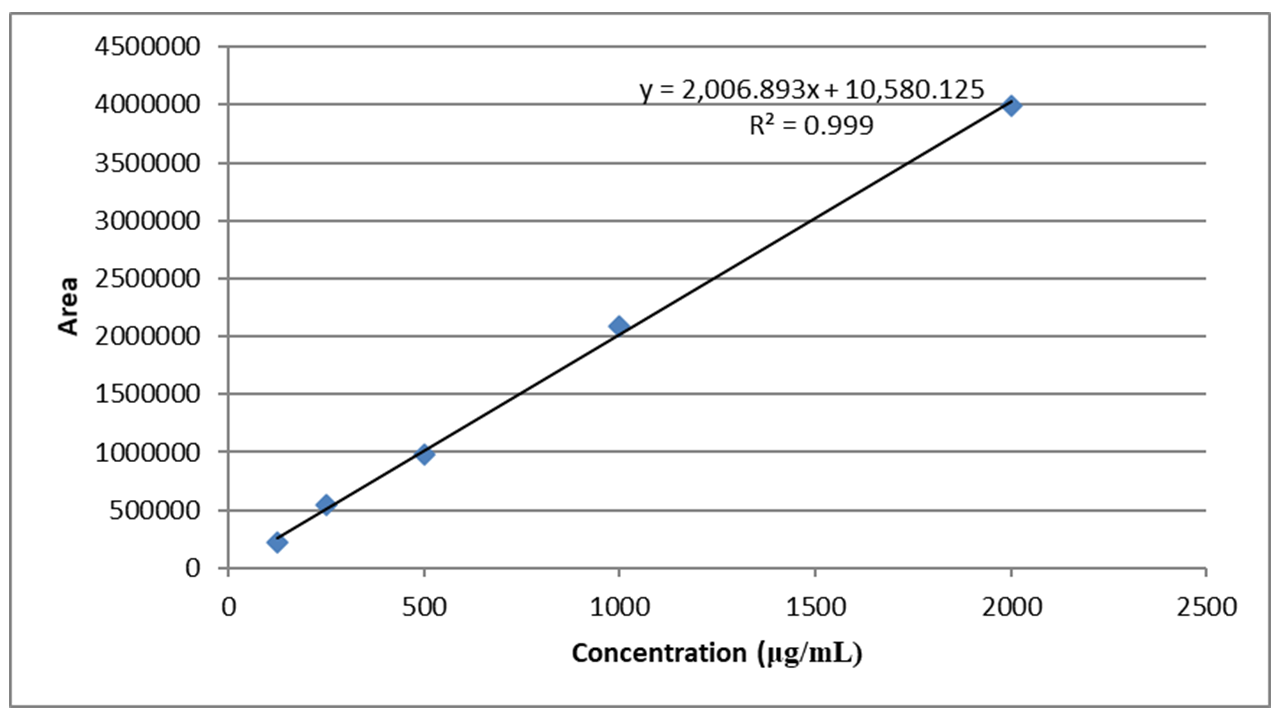


**Supplementary Figure 4.** **Calibration curve of different β-alanine concentrations by HPLC analysis.**


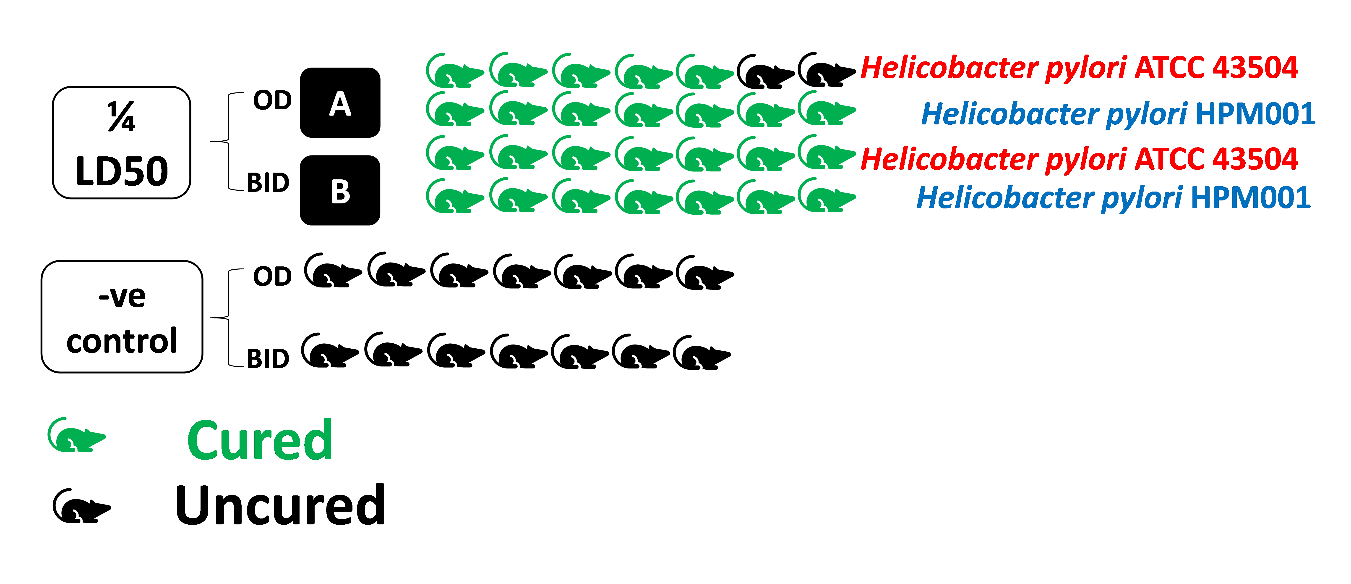


**Supplementary Figure 5.** **The outcome of malonic acid treatment regimens**

A diagrammatic representation of the number of rats that successfully cleared the infection (green) and failed to clear the infection (black) in each tested group by the end of the three weeks treatment period

**LD_50_:** Lethal dose 50; **OD:** Once daily; **BID:** Twice daily
